# Enhanced biomethane production from organic matter recovered from municipal wastewater by a pilot-scale plant continuous high-rate contact stabilization process

**DOI:** 10.1038/s41598-026-41598-w

**Published:** 2026-02-26

**Authors:** Kensuke Sakurai, Chika Abe

**Affiliations:** Innovative Materials and Resources Research Center, Public Works Research Institute, 1-6, Minamihara, Tsukuba, 305-8516 Ibaraki Japan

**Keywords:** Pilot-scale plant, Carbon recovery, Anaerobic digestion, Primary effluent, Municipal wastewater, High-rate contact stabilization, Engineering, Environmental sciences

## Abstract

High-rate contact stabilization (HiCS) processes for efficiently recovering organic matter from municipal wastewater are gaining attention as a means of producing renewable energy. In this study, a pilot-scale continuous HiCS process was applied to the effluent from a primary clarifier at a municipal wastewater treatment plant—a setting rarely reported in the literature, despite its importance for future full-scale implementation. The results confirmed that the continuous HiCS process recovers more organic matter than the conventional activated sludge (CAS) process while reducing organic matter oxidation. Biomethane potential testing further demonstrated that sludge recovered from the HiCS process generated methane at higher yields and faster rates compared to sludge from the CAS process. Moreover, the energy recovered per unit of removed organic matter in the HiCS process under practical operating conditions was higher than that achieved with the CAS process.

**Supplementary Information:**

The online version contains supplementary material available at 10.1038/s41598-026-41598-w.

## Introduction

The collection and treatment of municipal wastewater consume approximately 1% of the world’s electricity, and this share is expected to increase with the growing number of sewerage system users worldwide^1^. Reducing electricity consumption in the wastewater sector is therefore an important issue in addressing climate change^2,3^. Wastewater treatment plants (WWTPs) typically receive large amounts of organic matter through sewer networks.Improving the efficiency of organic matter recovery from wastewater and enhancing methane production from the recovered organics are expected to be effective strategies for increasing electricity generation and reducing net energy consumption^4^.

Many WWTPs have a primary clarifier, where settled organic matter is collected as primary sludge (PS). However, a substantial fraction of organic matter remains in the effluent from the primary clarifier. To efficiently recover this remaining organic matter as sludge and increase electricity generation, the high-rate contact stabilization (HiCS) process is considered promising^5,6^. The HiCS process promotes biological flocculation of organic matter in wastewater while suppressing its oxidation, enabling recovery of the organics by gravity sedimentation^7^. The recovered organics have a higher methane conversion rate than excess sludge (ES) produced in the conventional activated sludge (CAS) process^8,9^. This higher methane yield per unit of removed organic matter is one of the key advantages of the HiCS process. The HiCS process is a suspended-growth biological treatment system with a solids retention time (SRT) of less than 2 days, which limits excessive oxidation of organic matter compared to the CAS process, which typically has an SRT of 5 days or more. Sludge in the reaction tank is subjected to feast–famine conditions, promoting the formation of extracellular polymeric substances (EPS), which play a crucial role in biological flocculation^6,10^. Consequently, the methane recovery rate (MRR), normalized to the amount of organic matter removed in the HiCS process, was reported to be 0.24–0.29 g-COD/g-COD_rem_, which is higher than that of the CAS process (0.08–0.21 g-COD/g-COD_rem_)^9^.

Comparable effluent quality to that of the CAS process can be achieved when the HiCS process is followed by an activated sludge process, referred to as the HiCS–AS process^9^. The HiCS–AS process requires a larger footprint than the CAS process, but may be practical for full-scale WWTPs with sufficient land availability. By recovering additional methane from sludge produced in the secondary AS process, the HiCS–AS process has been shown to outperform the CAS process in terms of methane yield from the influent organic load^6,9^ and to achieve a higher net energy gain, even after accounting for additional energy consumption^6^.

There have been reports on a sequencing batch reactor (SBR)-based HiCS process for treating effluent from primary clarifiers^8,9,11^. However, reports on continuous operation are limited, even though this configuration is simpler to operate and maintain and could be more readily implemented in full-scale WWTPs.

Several studies have examined continuous HiCS processes applied to effluent from chemically enhanced primary treatment (CEPT)^10–12^, which may differ from primary effluent without coagulant addition, as CEPT effluent can contain residual iron ions^13–15^. Other cases have investigated the HiCS process without a primary clarifier in series^6,7^. In such cases, however, a large fraction of settleable organic matter may be oxidized, and the increased quantity of settleable material could influence sludge settleability and methane fermentability. Therefore, insights from systems without a primary clarifier are not directly applicable when evaluating the performance of the HiCS process treating primary effluent. For practical application, pilot-scale plant studies using primary effluent are valuable for an in-depth understanding of process performance. Because sludge produced by the HiCS process tends to settle more slowly than sludge produced by the CAS process despite the promotion of biological flocculation^10^, further investigation into the relationship between settling tank performance and organic matter recovery is warranted.

Methane fermentation characteristics of the recovered organic matter are also critical. However, no previous studies have examined the methane fermentation characteristics of sludge generated by a continuous HiCS process treating primary effluent. In the case of continuous HiCS operation for low-strength wastewater, previous studies using CEPT effluent did not investigate the methane fermentation characteristics of the recovered sludge^10–12^. Although the methane fermentation characteristics of sludge from the HiCS process without a primary clarifier have been studied^7^, such sludge contains large amounts of organic matter equivalent to PS and is therefore likely different from that generated by the HiCS process with a primary clarifier, as in the present study. For the SBR-based HiCS processes, Sakurai et al^8,9^. demonstrated that sludge generated from primary effluent produced substantially more methane than sludge from the CAS process, despite containing little organic matter equivalent to that which would settle in the primary clarifier. However, sludge from continuous and SBR operations may not share the same properties.

In summary, the continuous HiCS process appears to be a promising option for integration into full-scale WWTPs as the initial stage of the HiCS–AS process treating primary effluent without coagulant addition. Nevertheless, there is a lack of knowledge regarding its performance under practical operating conditions—knowledge essential for full-scale application. The objective of this study was to assess the performance of the continuous HiCS process as an energy recovery method by evaluating its organic matter recovery rate and the methane conversion efficiency of the recovered sludge.

## Methods

### Description of pilot plant operation

**Fig. 1:**
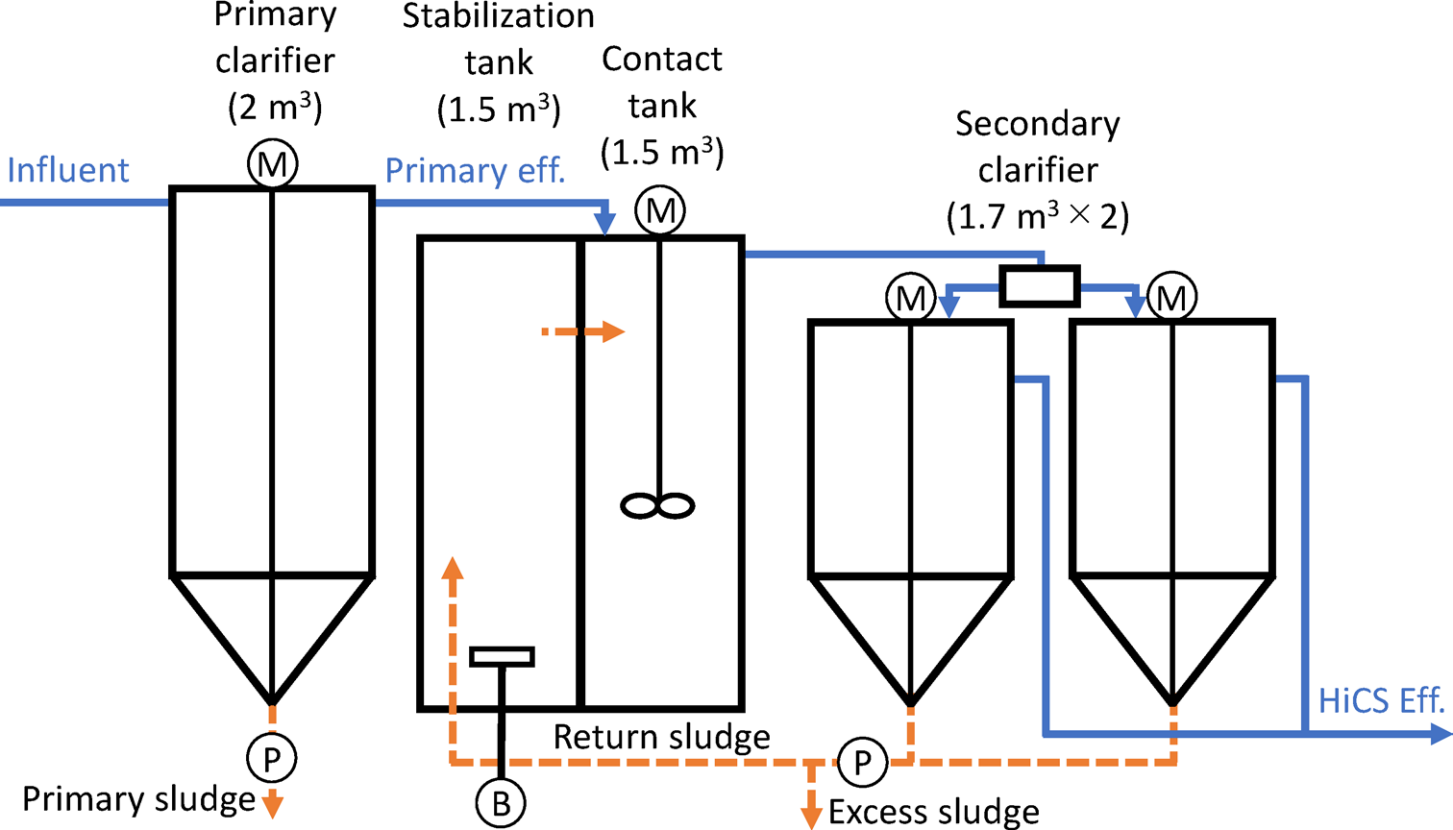
Schematic of the pilot-scale high-rate contact stabilization process (P: pump; B: blower; M: mixer).

As shown in Fig. 1, a pilot-scale plant consisting of a primary clarifier, stabilization tank, contact tank, and secondary clarifier was installed inside a building at the full-scale WWTP. Details of the pilot plant are provided in Table S1. The system was supplied with 28.8 m^3^/d of actual wastewater, taken after preliminary treatment at the full-scale WWTP. PS was withdrawn once per hour, totaling 0.24 m^3^/d, and the primary effluent was fed into the contact tank. The sludge return rate was maintained at 50% of the influent flow. To minimize the influence of seasonal temperature fluctuations, each operational period was defined as 11 consecutive days. The study was conducted over two periods, with different ES withdrawal rates. In Period 1, the ES withdrawal rate was 0.86 m^3^/d, and in Period 2, it was 1.72 m^3^/d. Changes in water temperature and sludge withdrawal volumes over time for each period are shown in Figure S1. The surface overflow rates in the primary and secondary clarifiers were 2.4 and 0.62 m^3^/(m^2^·h), respectively. In the stabilization tank, aeration was adjusted to maintain dissolved oxygen (DO) levels above 2.0 mg/L at all times, with an average of approximately 3.0 mg/L. The contact tank was not aerated, following the findings of Meerburg et al.^7,16^ that EPS production and bioflocculation are enhanced by the oxygen carried in from the stabilization tank. Consequently, DO levels in the contact tank remained below 0.5 mg/L throughout operation.

### Sampling and measurements

The influent, primary effluent, and effluent from the HiCS process were collected as composite samples. Each composite sample was prepared by mixing equal portions from 12 individual grab samples collected every 2 h over a 24-h period. The collected samples was kept refrigerated until analysis. Grab samples of ES and sludge from the contact tank were also collected. All samples were taken five times in Periods 1 and 2. Chemical oxygen demand (COD) was measured using COD reaction vials and a visible spectrophotometer (HACH Company, Loveland, CO). Soluble COD (sCOD), colloidal COD (cCOD), and particulate COD (pCOD) were determined. sCOD was measured using the method of Mamais et al.^17^, and cCOD was calculated by subtracting sCOD from the COD of samples filtered through glass microfiber filters (934-AH, Whatman). pCOD was calculated as the difference between total COD (tCOD) and cCOD. Total suspended solids (TSSs), volatile suspended solids (VSSs), and sludge volume index (SVI) were measured according to standard methods^18^. Loosely bound (LB)-EPS and tightly bound (TB)-EPS were determined using the method of Rahman et al^11^. Water temperature in the contact tank was continuously monitored, and ambient temperature data were obtained from the nearest Japan Meteorological Agency station in the same city^19^.

### Biomethane potential test

PS and ES generated from the HiCS pilot plant were collected during Period 2. Waste activated sludge (WAS) from an experimental plant using the CAS process with an SRT of 12 days, treating the same influent as the HiCS process, was also collected. ES from the HiCS process (HiCS sludge) generated during Period 1 was collected before the start of Period 2. All samples were concentrated and stored at 4 °C for 2–5 days prior to simultaneous biomethane potential (BMP) testing using a batch procedure. The BMP test was performed in triplicate for each substrate using an automated methane potential test system (AMPTS3; Bioprocess Control AB, Sweden). The inoculum consisted of digested sludge from a full-scale WWTP, pre-incubated at 35 °C for 1 week to reduce endogenous methane production. For each test, 0.8 L of inoculum was added to a 1 L glass vessel. Volatile solids (VS) and tCOD of each substrate were measured. For the inoculum, VS was 13.9 g/L, tCOD was 22.2 g/L, and pH was 7.8. To achieve an inoculum-to-substrate ratio (ISR, VS basis) of 5.0, each substrate was diluted with boiled, cooled tap water to 0.2 L and added to each vessel. An ISR of 4 or higher must be applied for easily degradable substrates^20^. A conservative value was adopted here as the optimal ISR for HiCS sludge has not been established, consistent with previous studies^8,9^. Vessels were incubated in a water bath at 35 °C with continuous stirring. The produced gas was passed through 3 M sodium hydroxide to remove carbon dioxide. Methane production was recorded for 28 days, with cumulative methane volumes logged hourly. Net methane production for each substrate was determined by subtracting the methane production in the blank assay from the total measured volume. Following Holliger et al.^21^, methane production was considered complete when the methane generation rate (excluding inoculum-derived methane) for three consecutive days before the end of the test was less than 1% of the cumulative volume. The methane yield at the end of the 28-day period was defined as the BMP. Microcrystalline cellulose (MCC; Sigma-Aldrich, #310697) was used as a positive control. All gas volumes were converted to standard conditions (0 °C and 101.325 kPa). The pH of the sludge was not adjusted before testing; the pH of the inoculum–substrate mixture was measured at the end of the test to confirm it exceeded 6.8^22^. VS was measured according to standard methods^18^.

### Calculations

The carbon recovery rate (CRR) was defined as the ratio of the COD mass in the ES to the COD mass in the influent. The SRT (days) was calculated using Eq. (1) because concentrations differed between the contact and stabilization tanks:

1 $$\:\begin{array}{c}SRT=\frac{{V}_{Con}\times\:{X}_{Con}+{V}_{Sta}\times\:{X}_{ES}}{{Q}_{ES}\times\:{X}_{ES}+{Q}_{Eff}\times\:{X}_{Eff}}\end{array}$$

where V_Con_ is the contact tank volume (L), X_Con_ is the TSS concentration in the contact tank (mg/L), V_Sta_ is the stabilization tank volume (L), X_ES_ is the TSS in ES (mg/L), Q_ES_ is the ES flow rate (L/d), Q_Eff_ is the HiCS effluent flow rate (L/d), and X_Eff_ is the TSS in HiCS effluent (mg/L). The organic loading rate (OLR) was calculated by dividing the daily influent COD mass by the combined volume of the contact and stabilization tanks.

The hydrolysis coefficient ($\:{k}_{h}$) for methane generation was obtained by fitting the temporal methane production data to a first-order reaction model (Eq. (2)), as described by Jensen et al.^23^. The first-order model is widely used to describe methane fermentation when particle decomposition is the rate-limiting step^24^.$\:\:{k}_{h}$ was estimated by nonlinear regression analysis using statistical software (SPSS Statistics, IBM Corp., NY, USA).

2 $$\:\begin{array}{c}M={M}_{0}\times\:\left(1-{e}^{-{k}_{h}t}\right)\:\end{array}$$

where M is the methane production at time *t* (NL/kg-VSS) and $\:{M}_{0}$ is the BMP (NL/kg-VSS). The coefficient of determination (R^2^) was calculated as one minus the ratio of the residual sum of squares to the adjusted total sum of squares. The COD-based methane conversion ratio (f_d_) was determined by converting methane production to COD using a factor of 0.35 NL-CH_4_/g-COD-CH_4_^9^ and dividing by the COD-to-VS ratio (COD/VS) of the substrate. The time required for 99% substrate conversion in a continuous stirred-tank reactor (CSTR), T_99_, was taken from the literature^25^.

The CRR, MRR were calculated following the equations reported by Sakurai et al^9^.. Following the methodology of Rahman et al.^12^, the carbon redirection rate (R) and the carbon harvesting rate (H) were computed using Eqs. (3) and (4) respectively:

3 $$\:\begin{array}{c}R=\:\frac{{Q}_{ES}\times\:({tCOD}_{ES}-{tCOD}_{Eff})+{(Q}_{Eff}+{Q}_{ES})\times\:{pCOD}_{Eff}}{{Q}_{In}\times\:{tCOD}_{In}}\end{array}$$

4 $$\:\begin{array}{*{20}{c}} {H = \frac{{{Q_{ES}} \times \:(tCO{D_{ES}} - tCO{D_{Eff}})}}{{{Q_{ES}} \times \:(tCO{D_{ES}} - tCO{D_{Eff}}) + ({Q_{Eff}} + {Q_{ES}}) \times \:pCO{D_{Eff}}}}} \end{array}$$

where, $\:{tCOD}_{ES}$ is the tCOD of the wasted sludge (mg/L), and $\:{tCOD}_{Eff}$ is the tCOD of the effluent (mg/L), $\:{pCOD}_{Eff}$ is the pCOD of the effluent (mg/L), *Q*_*In*_ is the influent flowrate(L/d), $\:{tCOD}_{In}$ is the tCOD of the influent (mg/L).

The CRR per unit of removed organic matter (CRR_rem_, g-COD/g-COD) and the corresponding MRR (MRR_rem_, g-COD-CH_4_/g-COD) were determined using Eqs. (5) and (6) given as follows:

5 $$\:\begin{array}{c}{CRR}_{rem}=\frac{CRR\times\:{tCOD}_{In}}{{tCOD}_{In}-{tCOD}_{Eff}}\end{array}$$

6 $$\:\begin{array}{c}{MRR}_{rem}=\frac{MRR\times\:{tCOD}_{In}}{{tCOD}_{In}-{tCOD}_{Eff}}\end{array}$$

where $\:{tCOD}_{In}$ and $\:{tCOD}_{Eff}$ are tCOD of the influent to and effluent from the HiCS process, respectively (mg-COD/L).

For comparison with standard CAS process values, CRR_rem_ for the CAS process was calculated using Eq. (5) after determining CRR as the product of the sludge conversion rate and sludge recovery rate, based on calculated parameters. The sludge redirection rate for the CAS process (R’) was calculated using Eqs. (7) and (8)^26^, applying representative parameter values reported in the literature^26^.

7 $$\:\begin{array}{c}{\mathrm{R}}^{{\prime\:}}=\frac{\left(1-{f}_{{S}^{{\prime\:}}us}-{f}_{{S}^{{\prime\:}}up}\right){Y}_{H}}{\left(1+{b}_{HT}SRT\right)}\cdot\:\left(1+{f}_{H}{b}_{HT}SRT\right)+{f}_{{S}^{{\prime\:}}up\:}\end{array}$$

8 $$\:\begin{array}{c}{b}_{HT}={b}_{H20}{\theta\:}^{\left(T-20\right)}\:\end{array}$$

where $\:{b}_{HT}$ is the endogenous respiration rate at T°C, unbiodegradable particulate and soluble organic matter ($\:{f}_{S{\prime\:}up}$ and $\:{f}_{S{\prime\:}us}$) are set at 0.04 and 0.12 (mg-COD/mg-COD), respectively, the yield coefficient ($\:{Y}_{H}$) is 0.67 (mg-COD/mg-COD), the endogenous respiration rate ($\:{b}_{H20}$) at 20 °C is 0.24 (1/d), and the temperature correction factor ($\:{\uptheta\:}$) is 1.029. The carbon harvesting rate for the CAS process was calculated assuming an effluent TSS of 10.0 mg/L and converting to pCOD using standard CAS process ratios (0.74 for VSS/TSS and 1.45 for pCOD/VSS)^27^, resulting in 10.7 mg-pCOD/L. MRR_rem_ for the CAS process was then obtained by multiplying the calculated CRR_rem_ by the BMP of WAS from the CAS process observed in this study. Additionally, in Eqs. (7) and (8), the main CAS process parameters (Y_H_ and b_H20_) were varied within their reported ranges to examine how strongly the HiCS process outperforms the CAS process. Based on references^26,28,29^, Y_H_ was varied from 0.38 to 0.75 and b_H20_ from 0.24 to 0.76; CRR_rem_ was recalculated for all combination of these values.

To test for significant differences in performance between periods, Welch’s t-test was performed using BellCurve 4.05 for Excel (Social Survey Research Information Co., Ltd., Tokyo, Japan).

## Results and discussion

### Water quality and properties of the pilot plant

**Fig. 2:**
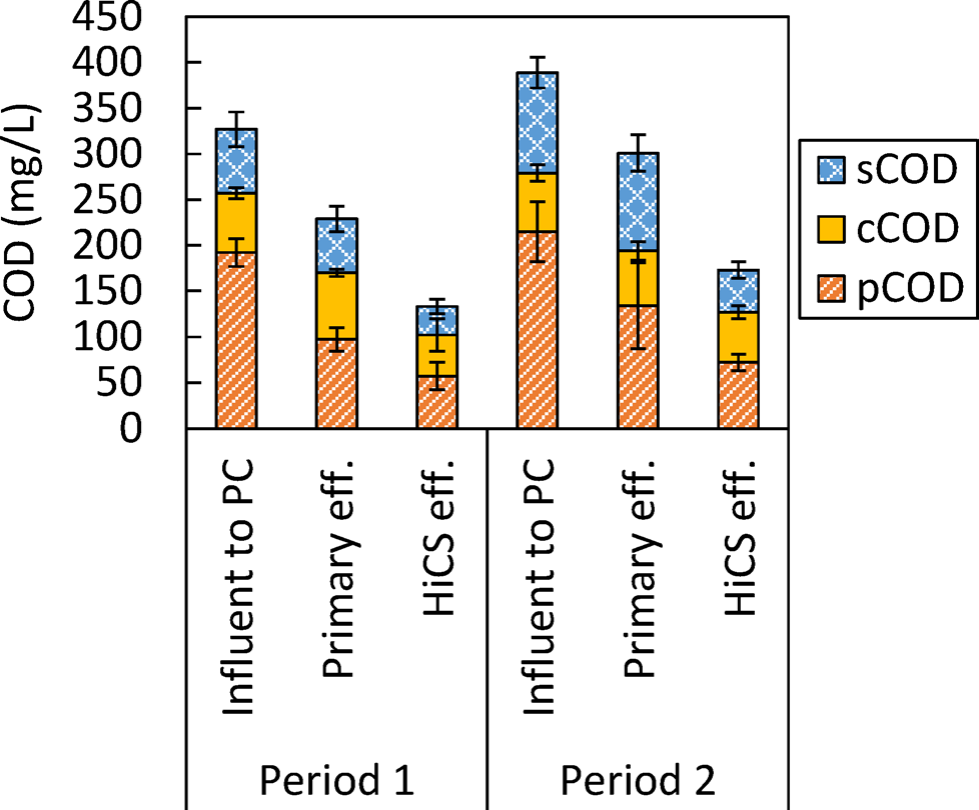
COD distribution for each form in the influent to the primary clarifier (PC), the primary effluent, and the effluent from the HiCS process during Periods 1 and 2. The primary effluent corresponds to the influent to the HiCS process.

Figure 2 shows the COD distribution for each form in the influent to the primary clarifier, the primary effluent (equivalent to the influent to the HiCS process), and the effluent from the HiCS process in Periods 1 and 2. The tCOD removal rates by the HiCS process were 0.43 ± 0.06 and 0.42 ± 0.10 g-COD/g-COD for Periods 1 and 2, respectively. Other water quality measurements are presented in Table S2.

Table 1 summarizes the sludge properties in the contact tank. In Periods 1 and 2, the TSS of the primary effluent was 33 ± 12 mg/L and 37 ± 12 mg/L, respectively, whereas the TSS in the reaction tank was 254 ± 40 mg/L and 123 ± 27 mg/L, respectively. The VSS/TSS ratios of sludge in the contact tank were 0.93 ± 0.02 and 0.91 ± 0.01 for Periods 1 and 2, respectively—substantially higher than the average value (0.74) typically reported for the CAS process^27^.

The total EPS content (sum of LB-EPS and TB-EPS) was comparable to that reported for a HiCS process with an SRT of 0.47 days and an average water temperature of 23°C^8^. Bioflocculation, primarily driven by EPS^30^, is considered to occur through the adsorption of colloidal and suspended organic carbon onto sludge via physicochemical effects^31^. Higher specific sCOD removal rates and higher food-to-microorganism (F/M) ratios tended to correspond with increased EPS production, consistent with previous findings^8,32^.

Although the SVI in Period 1 was slightly higher than in Period 2, no significant deterioration in sludge retention in the effluent was observed. It has been noted that SVI is not always reliable as an indicator of solid–liquid separation, particularly in high-rate systems, as it overemphasizes inhibition settling and compaction characteristics in settling tanks^33,34^. Due to the limited information currently available on SVI, further investigation is needed before it can be established as a reliable operational indicator for maintenance in such systems.

**Table 1:** Properties of sludge in the contact tank of the HiCS process.

	Unit	Period 1 (*n* = 5)	Period 2 (*n* = 5)
Ambient air temperature	°C	15 ± 2	17 ± 0
Contact tank water temperature	°C	18 ± 1	21 ± 0
SRT	d	0.98 ± 0.18	0.49 ± 0.11
OLR	kg-COD/(m^3^ཥd)	2.2 ± 0.2	2.9 ± 0.4
TSS	mg/L	254 ± 40	123 ± 27
VSS	mg/L	237 ± 34	112 ± 17
LB-EPS	mg-COD/g-VSS	70 ± 27	119 ± 20
TB-EPS	mg-COD/g-VSS	336 ± 26	352 ± 13
F/M ratio	g-sCOD/(g-VSSཥd)	1.4 ± 0.5	3.9 ± 1.2
Specific sCOD removal rate	g-sCOD_rem_/(g-VSSཥd)	0.71 ± 0.51	2.22 ± 0.62
SVI	mL/g-TSS	281 ± 42	166 ± 26

Note: Values after the ± sign are standard deviations. SRT was calculated using Eq. (1).

### Carbon recovery rate of the HiCS process

**Fig. 3:**
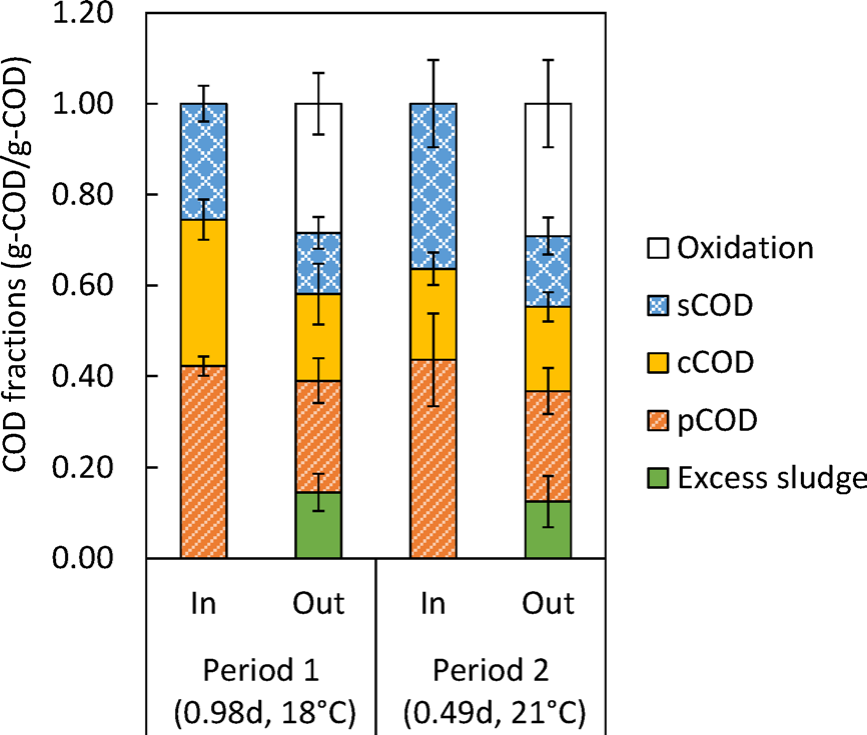
Fractions of the incoming and outgoing COD mass of the HiCS process in Periods 1 and 2. Error bars indicate standard deviations. Parentheses in the horizontal axis labels show the average SRT and water temperature.

The percentages of COD mass entering and leaving the HiCS process are shown in Fig. 3. The CRR, defined as the proportion of organic matter recovered as ES, from the HiCS process in Periods 1 and 2 was 0.15 ± 0.04 and 0.13 ± 0.06 g-COD/g-COD, respectively, relative to the influent COD mass. No significant differences were observed between the two periods despite variations in water temperature and SRT (Welch’s t-test, *p* = 0.54). The 95% confidence interval for the difference in means was [− 0.05, 0.09]. This study prioritized reducing seasonal variation, which resulted in a short investigation period. The limited amount of collectable data did not allow the complete exclusion of differences. Further investigation is therefore needed for an in-depth understanding of the effect of these operational differences on process performance.

Previous studies report that water temperature has little effect on CRR at short SRTs of 0.6–3 days^9,35^, suggesting that water temperature has a minor influence in high-rate processes. Rey-Martínez et al^36^. investigated a continuous high-rate AS (HRAS) process, similar to the HiCS process, with short SRT for primary effluent. Their results showed that at SRTs of 1.2 and 2.1 days at 18–22 °C, CRR values were approximately 0.04 and 0.11 g-COD/g-COD, respectively. The higher organic matter recovery observed in our study is likely attributable to the shorter SRT, which effectively suppressed oxidation of organic matter. In comparison, the CRR of a SBR-based HiCS process treating similar primary effluent with SRTs between 0.39 and 0.49 days was reported as 0.12 ± 0.03 g-COD/g-COD^8,9^, consistent with the results obtained here. The reported CRR for the HiCS process treating CEPT effluent was approximately 0.3 g-COD/g-COD^10–12^, which differs considerably from the present results, as mentioned in the Introduction.

The recovered organics per unit of removed organics in Periods 1 and 2 were 0.34 ± 0.10 and 0.31 ± 0.13 g-COD/g-COD_rem_, respectively. These values exceed the CRR_rem_ (0.17 g-COD/g-COD_rem_) reported for a continuous HRAS process (SRT 2.1 days, 18–22 °C) treating primary effluent^36^. For the SBR-based HiCS process treating similar primary effluent with SRTs between 0.39 and 0.49 days, CRR_rem_ values ranged from 0.31 to 0.44 g-COD/g-COD_rem_^9^, which are generally consistent with our findings. For comparison, the CRR_rem_ of the CAS process was calculated using Eq. (3) after determining CRR from the product of the carbon redirection rate and the carbon harvesting rate, as described in the Calculations section. At an SRT of 10 days and the same water temperatures as in Periods 1 and 2, the calculated CRR_rem_ for the CAS process was 0.29 g-COD-CH_4_/g-COD_rem_. The corresponding results from this study were 18% and 5.2% higher, respectively. These findings confirm that the HiCS process can recover more organic matter per unit of removed organic matter than the CAS process, while suppressing organic matter oxidation. Additionally, sensitivity analysis was conducted considering variations in microbial characteristics (Y_H_ and b_H20_) in the actual CAS process. When Y_H_ and b_H20_ were varied within the ranges specified in Methods section, the CRR_rem_ of the CAS process varied from 0.11 to 0.33. The measured values for the HiCS process in this study were frequently near the upper limit of this range or higher, confirming the high organic matter recovery potential of the HiCS process under practical operating conditions.

### Carbon redirection and carbon harvesting rates

Carbon redirection and carbon harvesting are key mechanisms that directly influence organic matter recovery^12,37^. In terms of carbon redirection, incoming particulate and colloidal organics are adsorbed and incorporated into sludge, while soluble organics are absorbed by microorganisms and utilized for growth and oxidation^37^. The carbon redirection rate represents the amount of sludge generated relative to the incoming COD mass and corresponds to the sludge discharged either as effluent or ES^26^. The carbon harvesting rate is the proportion of redirected sludge that is recovered through sedimentation. Sludge sedimentation characteristics are known to be one of the main factors limiting the carbon harvesting rate^37^.

The carbon redirection and harvesting rates obtained in this study are presented in Table 2. The carbon redirection rates were within the range of 0.35–0.39 g-COD/g-COD reported for an SBR-based HiCS process operated under very similar influent quality and water temperature^9^. While the SBR configuration generally offers superior solid–liquid separation performance compared to continuous flow systems—owing to the absence of inflows and outflows during sedimentation^38,39^ the carbon harvesting rates in this study were also comparable to the 0.32–0.38 g-COD/g-COD range reported for the SBR-based HiCS process^9^. This suggests that there was no substantial difference in sedimentation performance between the continuous and SBR configurations under the tested conditions. By contrast, in a study of the HiCS process treating CEPT effluent^11^, the carbon redirection and harvesting rates were 0.52 ± 0.09 and 0.58 ± 0.05 g-COD/g-COD, respectively—values that differ noticeably from those obtained in the present study. These higher values may be attributable to the presence of residual iron ions in the CEPT-treated water^13–15^.

**Table 2:** Carbon redirection and harvesting rates in Periods 1 and 2.

	Period 1	Period 2
Carbon redirection rate (g-COD/g-COD)	0.38 ± 0.07	0.35 ± 0.05
Carbon harvesting rate (g-COD/g-COD)	0.38 ± 0.08	0.35 ± 0.14
Carbon recovery rate (g-COD/g-COD)	0.15 ± 0.04	0.13 ± 0.06

Note: Values after the ± sign represent standard deviations.

### Biomethane production from the recovered organic matter

**Fig. 4:**
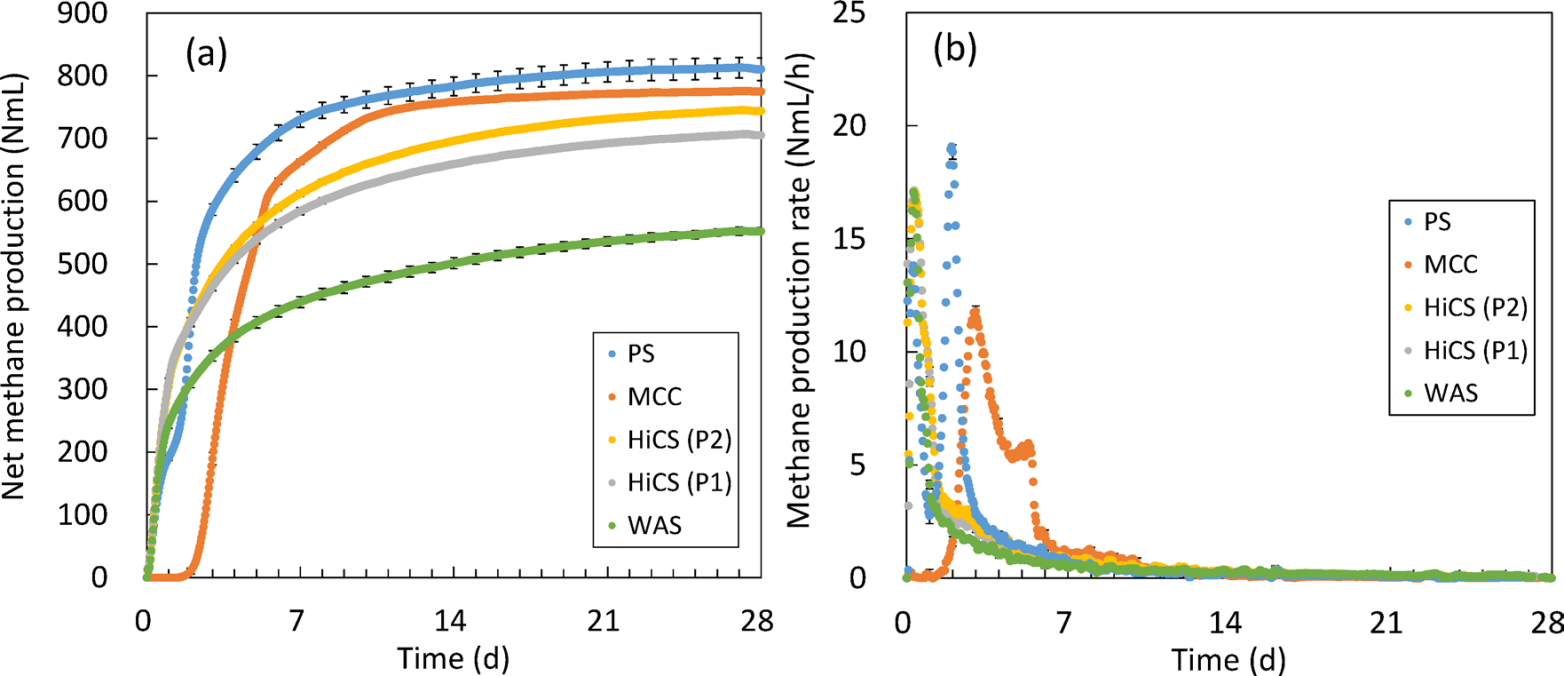
(**a**) Hourly cumulative methane production and (**b**) hourly methane production rate for each substrate. All tests were conducted in triplicate. Error bars represent daily standard deviations. MCC: microcrystalline cellulose; PS: primary sludge; WAS: waste activated sludge; HiCS (P1) and HiCS (P2): HiCS sludge from Periods 1 and 2, respectively.

The cumulative methane production for each substrate is shown in Fig. 4(a). The BMP of MCC, used as a positive control, was 358 ± 0.6 NL-CH_4_/kg-VS—almost identical to the value of 351 ± 5 NL-CH_4_/kg-VS reported in previous studies^40^. This falls within the MCC methane production criteria (340–395 NL-CH_4_/kg-VS) established by Holliger et al.^21^., indicating normal methane fermentation. The relative standard deviations for all substrates were low, with the highest being 2.2% for PS, meeting the 6% threshold^22^. The BMP of the blank assay was 33 NL-CH_4_/kg-VS, which was sufficiently low and within the inoculum criterion of 50 NL-CH_4_/kg-VS^20^.

A summary of the BMP tests for each substrate is provided in Table 3. Consistent with previous obsevations^8,9^, the BMP values for sludge from the HiCS process in Periods 1 and 2 were substantially higher than those of WAS from the CAS process. Moreover, methane production from HiCS sludge in both periods exceeded the BMP of sludge (276 NL-CH_4_/kg-VS) from a continuous HRAS process (SRT 1.2 days, water temperature 18–22 °C)^36^, which operated under conditions relatively similar to those of this study.

The BMP of HiCS sludge was higher during Period 2 than in Period 1, corresponding to an increase in water temperature and a decrease in SRT. In CAS processes, during the high-temperature period, increased endogenous respiration promotes the conversion of some readily biodegradable organic matter into slowly biodegradable substances^41^. A decrease in EPS, which serve as a substrate for methane production, has also been reported^42^. These factors could potentially reduce methane gas production^43^. However, because no clear changes in BMP were observed in previous study of the HiCS process^9^, the effect of reactor water temperature on BMP is considered to be minor. Ge et al.^44^. previously reported that in HRAS sludge with SRTs of 0.5–3 days, shorter SRTs leave more degradable organics, resulting in higher methane yields at 20–22 °C. Similar trends may apply to HiCS sludge, suggesting that the positive effect of residual degradable organic matter due to shortened SRT outweighed the possible negative impact of increased water temperature.

The BMP of HiCS sludge in this study was lower than the 387 NL-CH_4_/kg-VS reported for sludge from an SBR-based HRAS process (SRT 0.5 days, water temperature 20–22 °C) treating biofilm reactor effluent^44^, indicating potential for improvement. The BMP of PS was 0.75 g-COD-CH_4_/g-COD, consistent with previously reported values^45,46^, and considered representative.

The composition of organic matter—proteins, lipids, and carbohydrates—is related to the COD/VS ratio^27^. The COD/VS ratios of HiCS sludge in Periods 1 and 2 were 1.69 and 1.71, respectively, values close to those reported by Sakurai et al.^9^. for the SBR-based HiCS process at 22 °C. According to Ahnert et al.^27^, COD/VS ratios of PS and WAS are typically 1.8 ± 0.3 and 1.5 ± 0.2, respectively. In this study, the COD/VS ratio of PS was slightly lower, while that of WAS generally consistent with the reported average.

Hydrolysis coefficients decreased in the order: PS > HiCS sludge > WAS. The T_99_ of HiCS sludge was comparable to those of PS and WAS, indicating that it could achieve near-complete BMP yields within a standard anaerobic digester operating with an HRT of 20 days or more.

The methane production rate for each substrate is shown in Fig. 4(b). An enlarged view of the first 7 days is provided in Fig. S2. For HiCS sludge in Periods 1 and 2 and for WAS, the methane production rate peaked between 0.2 and 0.3 days after the start of the test. In contrast, PS exhibited two distinct peaks: the first at 0.2 days and the second at 2 days. This two-peak pattern is consistent with that reported by Yasui et al.^47^. It is estimated that 25%–30% of COD in municipal wastewater influent originates from toilet paper^48^, which is recovered in large quantities as PS^49^. Cellulose, the primary component of toilet paper, undergoes anaerobic digestion in which cellulolytic microorganisms produce enzymes that depolymerize cellulose into glucose. This glucose is subsequently fermented by cellulolytic and other glycolytic microorganisms^50^. Because of this process, the peak rate of cellulose-derived methanogenesis typically appears relatively late. In this study, MCC, used as a cellulose model, showed a methane production rate peak at day 2. Similarly, anaerobic digestion tests by Ghasimi et al.^40^ showed that methane production from toilet paper peaked approximately 1.9 days after the start of digestion. The second peak in the methane production rate observed for PS in this study closely matched the timing of the toilet paper-related peak reported in the literature, suggesting that it may be attributable to toilet paper degradation. In contrast, HiCS sludge from Periods 1 and 2 did not exhibit this second peak. This indicates that the large methane yields from HiCS sludge were not due to the slow decomposition of cellulose-rich material, but rather to more readily degradable organic matter.

**Table 3:** Summary of BMP testing for each substrate.

	MCC	HiCS sludge in P1	HiCS sludge in P2	PS	WAS
COD/VS ratio	1.11	1.69	1.71	1.38	1.56
BMP, $\:{M}_{0}$ (NL-CH_4_/kg-VS)	358 ± 1	317 ± 2	335 ± 2	363 ± 8	249 ± 2
BMP, $\:{f}_{d}$ (g-COD-CH_4_/g-COD)	0.92 ± 0.00	0.54 ± 0.00	0.56 ± 0.00	0.75 ± 0.02	0.45 ± 0.01
Hydrolysis coefficient, $\:{k}_{h}$ (/d)	0.19 ± 0.00	0.31 ± 0.00	0.29 ± 0.00	0.35 ± 0.00	0.27 ± 0.00
Coefficient of determination, R^2^	0.91	0.90	0.92	0.98	0.85
T_99_ (d)	24.0	14.8	15.6	13.1	16.9

Note: MCC: microcrystalline cellulose; PS: primary sludge; WAS: waste activated sludge from the CAS process; P1: Period 1; P2: Period 2. T_99_ is the time required for 99% conversion of the substrate to methane in a CSTR. Values following “±” represent standard deviations.

### Methane recovery rate by the HiCS process with anaerobic digestion

The MRRs of the HiCS process with anaerobic digestion in Periods 1 and 2 were 0.078 ± 0.022 and 0.070 ± 0.032 g-COD-CH_4_/g-COD, respectively. These correspond to electricity production values of 32 and 28 Wh/m^3^, respectively, assuming an organic concentration of 300 mg-COD/L in the primary effluent, a heat value of 13.9 kJ/g-COD-CH_4_ for methane^51^, and a gas engine power generation efficiency of 35%^4^. The MRR per unit of removed organic matter (MRR_rem_) for the HiCS process in Periods 1 and 2 was 0.18 ± 0.06 and 0.17 ± 0.07 g-COD-CH_4_/g-COD_rem_, respectively. The consistently high MRR_rem_ values over two periods demonstrate the HiCS process’s robustness against fluctuations in SRT and water temperature under practical conditions. For comparison, the MRR_rem_ of the CAS process was calculated by multiplying the CRR_rem_ (obtained as described above) by the BMP of WAS. At an SRT of 10 days and the same water temperatures as in Periods 1 and 2, the MRR_rem_ of the CAS process was 0.13 g-COD-CH_4_/g-COD_rem_. The HiCS process values in this study were 38% and 29% higher, respectively, confirming the high MRR_rem_ characteristic of the HiCS process. These results suggest that the continuous HiCS process enables efficient recovery of renewable energy even from primary effluent. By integrating the continuous HiCS process to full-scale WWTPs using the CAS process—converting them to a HiCS–AS system—the amount of generated renewable energy can be further increased through additional recovering of the organic matter remaining in the HiCS effluent.

## Conclusions

A pilot-scale HiCS process plant was operated over two periods with different SRTs and water temperatures, using primary effluent from actual wastewater. The efficiencies of organic matter recovery and methane conversion of the recovered organic matter were evaluated. In Period 1 (SRT 0.98 days, water temperature 18 °C), the organic matter recovery and methane conversion rates were 0.15 g-COD/g-COD and 0.54 g-COD/g-COD, respectively. In Period 2 (SRT 0.49 days, water temperature 21 °C), the corresponding values were 0.13 g-COD/g-COD and 0.56 g-COD/g-COD, respectively. In both periods, the methane conversion rates of the HiCS process were considerably higher than that of WAS from the CAS process (0.45 g-COD/g-COD). Methane production rate profiles showed that the high methane generation from HiCS sludge was not attributable to slowly decomposing organic matter with a day-2 peak, characteristic of PS. The hydrolysis coefficient of HiCS sludge was between those of PS and WAS from the CAS process. The MRR_rem_ of the HiCS process with anaerobic digestion was 0.18 ± 0.06 and 0.17 ± 0.07 g-COD-CH_4_/g-COD_rem_ for Periods 1 and 2, respectively, showing consistently high values in both periods, which indicates high robustness against fluctuations in SRT and water temperature in actual environments. Based on these results, integrating the continuous HiCS process into full-scale WWTPs using the CAS process is considered a promising approach to increase renewable energy generation.

## Supplementary Information

Below is the link to the electronic supplementary material.

Supplementary Material 1

## Data Availability

The datasets generated during and/or analyzed during the current study are available from the corresponding author on reasonable request.

## References

[1] International Energy Agency. *Water energy nexus (Excerpt from the World Energy Outlook)*, (2016). https://www.iea.org/reports/water-energy-nexus

[2] Zhang, X. & Liu, Y. Resource recovery from municipal wastewater: A critical paradigm shift in the post era of activated sludge. *Bioresour. Technol.* **363**, 127932. 10.1016/j.biortech.2022.127932 (2022). 36096327 10.1016/j.biortech.2022.127932

[3] Guven, H., Ersahin, M. E., Ozgun, H., Ozturk, I. & Koyuncu, I. Energy and material refineries of future: Wastewater treatment plants. *J. Environ. Manage.* **329**, 117130. 10.1016/j.jenvman.2022.117130 (2023). 36571955 10.1016/j.jenvman.2022.117130

[4] McCarty, P. L., Bae, J. & Kim, J. Domestic wastewater treatment as a net energy producer–can this be achieved?. *Environ. Sci. Technol.* **45**, 7100–7106. 10.1021/es2014264 (2011). 21749111 10.1021/es2014264

[5] Rahman, A. et al. Moving forward with A-stage and high-rate contact-stabilization for energy efficient water resource recovery facility: Mechanisms, factors, practical approach, and guidelines. *J. Water Process Eng.* **36**, 101329. 10.1016/j.jwpe.2020.101329 (2020).

[6] Rahman, A. et al. A-stage and high-rate contact-stabilization performance comparison for carbon and nutrient redirection from high-strength municipal wastewater. *Chem. Eng. J.* **357**, 737–749. 10.1016/j.cej.2018.09.206 (2019).

[7] Meerburg, F. A. et al. Toward energy-neutral wastewater treatment: A high-rate contact stabilization process to maximally recover sewage organics. *Bioresour. Technol.* **179**, 373–381. 10.1016/j.biortech.2014.12.018 (2015). 25553568 10.1016/j.biortech.2014.12.018

[8] Sakurai, K. & Okayasu, Y. Improvement of organic carbon recovery from wastewater by changing water temperature in high-rate contact stabilization process. *Int. J. Environ. Sci. Technol.* **22**, 2127–2136. 10.1007/s13762-024-05791-6 (2025).

[9] Sakurai, K., Okayasu, Y. & Abe, C. Energy recovery from organic matter in municipal wastewater using a two-stage system with high-rate contact stabilization and activated sludge processes under seasonal water temperature variations. *Environ. Sci. Water Res. Technol.* **11**, 1016–1025. 10.1039/d4ew00820k (2025).

[10] Van Winckel, T. et al. Enhancing bioflocculation in high-rate activated sludge improves effluent quality yet increases sensitivity to surface overflow rate. *Chemosphere* **308**, 136294. 10.1016/j.chemosphere.2022.136294 (2022). 36084824 10.1016/j.chemosphere.2022.136294

[11] Rahman, A. et al. Impact of aerobic famine and feast condition on extracellular polymeric substance production in high-rate contact stabilization systems. *Chem. Eng. J.* **328**, 74–86. 10.1016/j.cej.2017.07.029 (2017).

[12] Rahman, A. et al. Bioflocculation management through high-rate contact-stabilization: A promising technology to recover organic carbon from low-strength wastewater. *Water Res.* **104**, 485–496. 10.1016/j.watres.2016.08.047 (2016). 27589209 10.1016/j.watres.2016.08.047

[13] Checa-Fernández, A. et al. Direct application of chemically enhanced primary treatment in a municipal wastewater treatment plant: A case study. *Chem. Eng. Res. Des.* **204**, 183–192. 10.1016/j.cherd.2024.02.039 (2024).

[14] Lees, E. J., Noble, B., Hewitt, R. & Parsons, S. A. The impact of residual coagulant on the respiration rate and sludge characteristics of an activated microbial biomass. *Process Saf. Environ. Prot.* **79**, 283–290. 10.1205/095758201753189721 (2001).

[15] Lees, E. J., Noble, B., Hewitt, R. & Parsons, S. A. The impact of residual coagulant on downstream treatment processes. *Environ. Technol.* **22**, 113–122. 10.1080/09593332208618316 (2001). 11286051 10.1080/09593332208618316

[16] Meerburg, F. A., Boon, N., Van Winckel, T., Pauwels, K. T. G. & Vlaeminck, S. E. Live fast, die young: Optimizing retention times in high-rate contact stabilization for maximal recovery of organics from wastewater. *Environ. Sci. Technol.* **50**, 9781–9790. 10.1021/acs.est.6b01888 (2016). 27480015 10.1021/acs.est.6b01888

[17] Mamais, D., Jenkins, D. & Pitt, P. A rapid physical-chemical method for the determination of readily biodegradable soluble COD in municipal wastewater. *Water Res.* **27**, 195–197. 10.1016/0043-1354(93)90211-Y (1993).

[18] American Public Health Association, American Water Works Association & Water Environment Federation. Standard methods for the examination of water and wastewater. 23rd edition edn. American public health association, (2017).

[19] Japan Meteorological Agency. *Climate Statistics*, (2025). http://www.jma.go.jp/jma/index.html

[20] Holliger, C. et al. Towards a standardization of biomethane potential tests. *Water Sci. Technol.* **74**, 2515–2522. 10.2166/wst.2016.336 (2016). 27973356 10.2166/wst.2016.336

[21] Holliger, C. et al. Towards a standardization of biomethane potential tests: A commentary. *Water Sci. Technol.* **83**, 247–250. 10.2166/wst.2020.569 (2021). 33460422 10.2166/wst.2020.569

[22] VDI 4630. *Fermentation of organic materials - Characterization of the substrate, sampling, collection of material data, fermentation tests* (Engl. VDI-Gesellschaft Energie und Umwelt, 2016).

[23] Jensen, P. D., Ge, H. & Batstone, D. J. Assessing the role of biochemical methane potential tests in determining anaerobic degradability rate and extent. *Water Sci. Technol.* **64**, 880–886. 10.2166/wst.2011.662 (2011). 22097074 10.2166/wst.2011.662

[24] Batstone, D. J. et al. The IWA anaerobic digestion model no 1 (ADM1). *Water Sci. Technol.* **45**, 65–73. 10.2166/wst.2002.0292 (2002). 12188579

[25] Maldonado-Saeteros, S., Baquerizo-Crespo, R. J., Gómez-Salcedo, Y., Pérez-Ones, O. & Pereda-Reyes, I. Influence of temperature on kinetics and hydraulic retention time in discontinuous and continuous anaerobic systems. *Environ. Eng. Res.* **28**, 210442–210440. 10.4491/eer.2021.442 (2022).

[26] Ekama, G. A. & Wentzel, M. C. IWA Publishing, Organic matter removal, in *Biological Wastewater Treatment: Principles, Modelling and Design* (eds Guanghao Chen, Mark C. M. van Loosdrecht, George A. Ekama, & Damir Brdjanovic) 111–160 (2023).

[27] Ahnert, M. et al. Organic matter parameters in WWTP - A critical review and recommendations for application in activated sludge modelling. *Water Sci. Technol.* **84**, 2093–2112. 10.2166/wst.2021.419 (2021). 34810300 10.2166/wst.2021.419

[28] Lavallée, B., Lessard, P. & Besser, C. Decay rate variability of active heterotrophic biomass. *Water Sci. Technol.* **46**, 423–430. 10.2166/wst.2002.0511 (2002). 12216661

[29] Sharifi, S., Murthy, S., Takács, I. & Massoudieh, A. Probabilistic parameter estimation of activated sludge processes using markov chain monte carlo. *Water Res.* **50**, 254–266. 10.1016/j.watres.2013.12.010 (2014). 24384542 10.1016/j.watres.2013.12.010

[30] Sheng, G.-P., Yu, H.-Q. & Li, X.-Y. Extracellular polymeric substances (EPS) of microbial aggregates in biological wastewater treatment systems: A review. *Biotechnol. Adv.* **28**, 882–894. 10.1016/j.biotechadv.2010.08.001 (2010). 20705128 10.1016/j.biotechadv.2010.08.001

[31] Guthi, R. S. et al. A-Stage process - Challenges and drawbacks from lab to full scale studies: A review. *Water Res.* **226**, 119044. 10.1016/j.watres.2022.119044 (2022). 36272198 10.1016/j.watres.2022.119044

[32] Jimenez, J. et al. High-rate activated sludge system for carbon management -Evaluation of crucial process mechanisms and design parameters. *Water Res.* **87**, 476–482. 10.1016/j.watres.2015.07.032 (2015). 26260539 10.1016/j.watres.2015.07.032

[33] Mancell-Egala, W. et al. Novel stokesian metrics that quantify collision efficiency, floc strength, and discrete settling behavior. *Water Sci. Technol.* **89**, 586–597. 10.2175/106143017X14902968254494 (2017). 10.2175/106143017X14902968254494 28641670

[34] Mancell-Egala, W. et al. Limit of stokesian settling concentration characterizes sludge settling velocity. *Water Res.* **90**, 100–110. 10.1016/j.watres.2015.12.007 (2016). 26724444 10.1016/j.watres.2015.12.007

[35] Shao, Y. et al. Effect of operating temperature on the efficiency of ultra-short-sludge retention time activated sludge systems. *Environ. Sci. Pollut. Res.* **28**, 39257–39267. 10.1007/s11356-021-13481-w (2021). 10.1007/s11356-021-13481-w 33751351

[36] Rey-Martínez, N., Barreiro-López, A., Guisasola, A. & Baeza, J. A. Comparing continuous and batch operation for high-rate treatment of urban wastewater. *Biomass Bioenergy* **149**, 106077. 10.1016/j.biombioe.2021.106077 (2021).

[37] Canals, J. et al. High-rate activated sludge at very short SRT: Key factors for process stability and performance of COD fractions removal. *Water Res.* **231**, 119610. 10.1016/j.watres.2023.119610 (2023). 36680828 10.1016/j.watres.2023.119610

[38] U.S. EPA. *Wastewater technology fact sheet; Sequencing Batch Reactors*, (1999). https://www.epa.gov/system/files/documents/2022-10/sequencing-batch-reactors-factsheet.pdf

[39] Gerardi, M. H. SBR cycles, in *Troubleshooting the Sequencing Batch Reactor.* 17–28 (John Wiley & Sons, Inc., (2010).

[40] Ghasimi, D. S. M., Zandvoort, M. H., Adriaanse, M., van Lier, J. B. & de Kreuk, M. Comparative analysis of the digestibility of sewage fine sieved fraction and hygiene paper produced from virgin fibers and recycled fibers. *Waste Manag.* **53**, 156–164. 10.1016/j.wasman.2016.04.034 (2016). 27172811 10.1016/j.wasman.2016.04.034

[41] Henze, M., Gujer, W., Mino, T. & van Loosedrecht, M. *Activated sludge models ASM1, ASM2, ASM2d and ASM3* (IWA Publishing, 2006).

[42] Xie, F. et al. Seasonal temperature effects on EPS composition and sludge settling performance in full-scale wastewater treatment plant: Mechanisms and mitigation strategies. *Fermentation* **11**, 532. 10.3390/fermentation11090532 (2025).

[43] Appels, L., Baeyens, J., Degrève, J. & Dewil, R. Principles and potential of the anaerobic digestion of waste-activated sludge. *Prog. Energy Combust. Sci.* **34**, 755–781. 10.1016/j.pecs.2008.06.002 (2008).

[44] Ge, H., Batstone, D. J., Mouiche, M., Hu, S. & Keller, J. Nutrient removal and energy recovery from high-rate activated sludge processes - Impact of sludge age. *Bioresour. Technol.* **245**, 1155–1161. 10.1016/j.biortech.2017.08.115 (2017). 28863992 10.1016/j.biortech.2017.08.115

[45] Wei, W. et al. Sludge incineration bottom ash enhances anaerobic digestion of primary sludge toward highly efficient sludge anaerobic codigestion. *ACS Sustainable Chem. Eng.* **8**, 3005–3012. 10.1021/acssuschemeng.0c00015 (2020).

[46] Mahdy, A., Mendez, L., Ballesteros, M. & Gonzalez-Fernandez, C. Algaculture integration in conventional wastewater treatment plants: Anaerobic digestion comparison of primary and secondary sludge with microalgae biomass. *Bioresour. Technol.* **184**, 236–244. 10.1016/j.biortech.2014.09.145 (2015). 25451781 10.1016/j.biortech.2014.09.145

[47] Yasui, H., Goel, R., Li, Y. Y. & Noike, T. Modified ADM1 structure for modelling municipal primary sludge hydrolysis. *Water Res.* **42**, 249–259. 10.1016/j.watres.2007.07.004 (2008). 17719077 10.1016/j.watres.2007.07.004

[48] Ruiken, C. J., Breuer, G., Klaversma, E., Santiago, T. & van Loosdrecht, M. C. Sieving wastewater-cellulose recovery, economic and energy evaluation. *Water Res.* **47**, 43–48. 10.1016/j.watres.2012.08.023 (2013). 23121895 10.1016/j.watres.2012.08.023

[49] Tochioka, E. et al. Enhancing the dewaterability of anaerobically digested sludge using fibrous materials recovered from primary sludge: Demonstration from a field study. *Clean Technol. Environ. Policy* **21**, 1131–1141. 10.1007/s10098-019-01698-w (2019).

[50] Schwarz, W. The cellulosome and cellulose degradation by anaerobic bacteria. *Appl. Microbiol. Biotechnol.* **56**, 634–649. 10.1007/s002530100710 (2001). 11601609 10.1007/s002530100710

[51] Heidrich, E. S., Curtis, T. P. & Dolfing, J. Determination of the internal chemical energy of wastewater. *Environ. Sci. Technol.* **45**, 827–832. 10.1021/es103058w (2011). 21142001 10.1021/es103058w

