# Supplementary material for: Enhanced biomethane production from organic matter recovered from municipal wastewater by a pilot-scale plant continuous high-rate contact stabilization process
Source: Sci Rep. 2026 Feb 26;16:11078. doi: 10.1038/s41598-026-41598-w (PMC13043695; doi:10.1038/s41598-026-41598-w)
Supplement: Supplementary file 1 — Supplementary Material 1 [file 41598_2026_41598_MOESM1_ESM.docx]

**Supplementary Information**

**Enhanced biomethane production from organic matter recovered from municipal wastewater by a pilot-scale plant using continuous high-rate contact stabilization process**

Kensuke Sakurai ^1,*^ and Chika Abe ^1^

^1^ Innovative Materials and Resources Research Center, Public Works Research Institute, 1-6, Minamihara, Tsukuba, Ibaraki 305-8516, Japan

* Corresponding author: recycle-imarrc21@pwri.go.jp

**Table S1.** Hydraulic characteristics of sedimentation and reaction tanks.

|  | Unit | Primary clarifier | Secondary clarifier | Contact tank | Stabilization tank |
| --- | --- | --- | --- | --- | --- |
| Shape | － | Circular | Circular | Rectangular | Rectangular |
| Injected location | － | Center　feed-well | Center feed-well | Side of the tank | Side of the tank |
| Number of tanks | － | 1 | 2 | 1 | 1 |
| Effective volume | m^3^ | 2 | 1.7 | 1.5 | 1.5 |
| Tank size | m | Diameter:  1.1 | Diameter:  1.4 | 1.0 × 0.6 | 1.0 × 0.6 |
| Effective depth | m | 3.1 | 2.3 | 2.5 | 2.5 |
| Clarifier surface area | m^2^ | 0.50 | 0.97 | － | － |
| SOR relative to total inflow | m^3^  /(m^2^･h) | 2.4 | 0.62 | － | － |
| HRT relative to total inflow | h | 1.7 | 2.9 | 1.3 | 1.3 |

Note: SOR = surface overflow rate; HRT was calculated as the effective volume divided by the total inflow.

**Table S2.** Characteristics of influent and treated water in Periods 1 and 2.

| Parameter | Unit | Period 1 (n = 5) | | | Period 2 (n = 5) | | |
| --- | --- | --- | --- | --- | --- | --- | --- |
|  |  | Influent | Primary effluent | HiCS effluent | Influent | Primary effluent | HiCS effluent |
| tCOD | mg/L | 327 ± 11 | 230 ± 23 | 132 ± 23 | 389 ± 39 | 300 ± 42 | 172 ± 11 |
| pCOD | mg/L | 192 ± 15 | 97 ± 13 | 57 ± 15 | 215 ± 33 | 134 ± 47 | 72 ± 9 |
| cCOD | mg/L | 65 ± 6 | 73 ± 4 | 45 ± 18 | 64 ± 9 | 60 ± 10 | 55 ± 7 |
| sCOD | mg/L | 70 ± 19 | 59 ± 14 | 31 ± 8 | 110 ± 17 | 107 ± 20 | 46 ± 9 |
| TSS | mg/L | 92 ± 21 | 33 ± 12 | 30 ± 7 | 130 ± 26 | 37 ± 12 | 37 ± 10 |
| VSS | mg/L | 86 ± 21 | 30 ± 10 | 26 ± 6 | 118 ± 20 | 33 ± 11 | 33 ± 8 |

Note: Values after the ± sign are standard deviations.

**Figure S1.** Daily temperature and flow rate in Periods 1 and 2. AP indicates the acclimation period. Circled days indicated the sampling dates.

**Figure S2.** Enlarged view of Figure 1 for the first 7 days: (a) hourly cumulative methane production; (b) hourly methane production rate for each substrate. All substrates were tested in triplicate. Error bars represent daily standard deviations. MCC: microcrystalline cellulose; PS: primary sludge; WAS: waste activated sludge; HiCS (P1) and HiCS (P2): HiCS sludge from Periods 1 and 2.
